# Loss of immunometabolic adaptability in MASH: gut-derived signals drive macrophage reprogramming and fibrosis

**DOI:** 10.3389/fimmu.2026.1793092

**Published:** 2026-04-13

**Authors:** Yan Li, Yuyuan Hu, Yuan He, Yuhang Yang, Dingwen Xue, Erkui Xue, Jinghan Jia, Wei Zhang, Jinxi Wang

**Affiliations:** 1 Neurology, Third Hospital of Shanxi Medical University, Shanxi Bethune Hospital, Shanxi Academy of Medical Sciences Tongji Shanxi Hospital, Taiyuan, China; 2 Division of Colorectal Surgery, Third Hospital of Shanxi Medical University, Shanxi Bethune Hospital, Shanxi Academy of Medical Sciences Tongji Shanxi Hospital, Taiyuan, China; 3 Hepatobiliary Surgery, Baogang Hospital of Inner Mongolia, Baotou, China; 4 Yuncheng Central Hospital affiliated to Shanxi Medical University, Yuncheng, Shanxi, China

**Keywords:** gut–liver axis, immunometabolism, inflammasome activation, macrophage metabolic reprogramming, metabolic dysfunction–associated steatohepatitis (MASH)

## Abstract

Metabolic dysfunction–associated steatohepatitis (MASH) is a progressive inflammatory subtype of metabolic dysfunction–associated steatotic liver disease (MASLD), characterized by hepatocellular steatosis, persistent inflammation, and varying degrees of fibrosis. Although multiple therapeutic strategies targeting inflammatory or metabolic pathways have entered clinical development, their overall efficacy remains limited, suggesting that the mechanisms driving sustained disease progression remain incompletely understood. Previous studies have largely focused on inflammatory cascades, whereas the role of immune cell energy metabolism in sustaining inflammation and promoting fibrosis has received comparatively less attention. Recent work has increasingly shifted toward immunometabolic reprogramming, indicating that metabolic signals derived from the gut microbiota may contribute to the establishment and maintenance of the hepatic immune microenvironment. In this context, reductions in short-chain fatty acids and secondary bile acids, together with increased succinate and endotoxin levels, may alter the energy metabolism of Kupffer cells and infiltrating macrophages through signaling pathways involving FXR/TGR5 and mTOR/AMPK, thereby favoring a pro-inflammatory phenotype. This metabolic shift is associated with enhanced inflammatory signaling linked to HIF-1α, increased NLRP3 inflammasome activity, and paracrine effects that may promote hepatic stellate cell activation during fibrotic progression. Overall, current evidence supports a model in which MASH progression is associated with a gradual loss of immunometabolic adaptability in the setting of metabolic dysregulation along the gut–liver axis. Reduced metabolic flexibility may limit the ability of immune cells to transition between functional states, thereby hindering resolution of inflammation and contributing to pathological tissue remodeling. Within this framework, single-target interventions may be insufficient to fully restore immunometabolic homeostasis, whereas strategies that concurrently address gut microbial function and key metabolic signaling pathways may be more mechanistically sound. Considering MASH as a model of systemic immunometabolic dysregulation may also provide insight into other metabolism-associated inflammatory diseases, although extrapolation should remain cautious.

## 1 Introduction

Metabolic dysfunction–associated steatotic liver disease (MASLD) has become one of the most prevalent chronic liver diseases worldwide, with its rising incidence closely linked to the increasing prevalence of obesity and metabolic syndrome (1). Within the disease spectrum of MASLD, metabolic dysfunction–associated steatohepatitis (MASH) is characterized by steatosis accompanied by persistent inflammatory responses and varying degrees of fibrosis, representing a critical stage in progression toward cirrhosis and hepatocellular carcinoma (2). Although numerous candidate therapeutic strategies targeting inflammatory or metabolic pathways have entered clinical investigation in recent years, their overall efficacy remains limited, with substantial interindividual variability in treatment response (3). These observations suggest that interventions targeting a single inflammatory or metabolic pathway may be insufficient to effectively halt disease progression in the majority of patients (4).

The gut–liver axis has thus increasingly attracted attention as a critical pathway linking host metabolic homeostasis with immune regulation (5). Early studies primarily focused on impairment of the intestinal mucosal barrier induced by gut microbiota dysbiosis and subsequent endotoxin translocation, identifying lipopolysaccharide and other pathogen-associated molecular patterns (PAMPs) as key triggers of hepatic innate immune activation (6). With advances in this field, the role of the gut microbiota in MASH is no longer considered to be restricted to the single mechanism of increased endotoxin burden (7). The gut microbial ecosystem itself exhibits substantial metabolic activity, generating short-chain fatty acids, modulating bile acid composition, and influencing indole compounds derived from tryptophan metabolism, thereby providing sustained metabolic signaling inputs to the liver (8).

These gut-derived metabolic signals can act through sensing pathways such as the farnesoid X receptor (FXR), Takeda G protein–coupled receptor 5 (TGR5), the aryl hydrocarbon receptor (AhR), and mTOR/AMPK to influence the transcriptional programs and metabolic states of hepatic immune and parenchymal cells, thereby contributing to the regulation of the threshold for activation or resolution of local inflammatory responses (9). In contrast to acute inflammatory stimuli, these metabolic cues typically exert low-grade and persistent effects on the hepatic immune system, and are therefore more likely to reshape the basal regulatory state of immune responses rather than directly triggering overt acute inflammation (10).

Notably, multiple studies have reported that aberrant metabolic signaling frequently coexists with immune homeostatic imbalance in MASH, as exemplified by altered Th17/Treg ratios and enhanced pro-inflammatory functions of macrophages (11). However, most existing reviews tend to regard metabolic dysregulation and immune activation as relatively independent or sequential pathological processes, with limited systematic analysis of their intrinsic interconnection from the perspective of the intrinsic metabolic states of immune cells (12). With advances in the field of immunometabolism, accumulating evidence indicates that the functional phenotypes of immune cells are tightly coupled to their intracellular energy metabolic programs (13). For example, within specific metabolic microenvironments, hepatic macrophages can shift from an oxidative phosphorylation–dominant metabolic profile to a predominantly glycolytic state; such metabolic reprogramming facilitates sustained production of pro-inflammatory mediators and may reduce sensitivity to negative feedback regulatory signals (14).

On this basis, descriptions limited to changes in microbial composition or individual metabolite levels are no longer sufficient to explain the marked heterogeneity of MASH in terms of inflammatory severity, the rate of fibrotic progression, and therapeutic responsiveness (15). Although the relationship between metabolic dysregulation and immune activation has received considerable attention, studies that systematically examine their intrinsic connection from the perspective of the metabolic states of immune cells remain relatively limited.

Accordingly, this review adopts the perspective of immunometabolic reprogramming to systematically examine how metabolic signals derived from the gut microbiota contribute to the maintenance of inflammation and the progression of fibrosis by reshaping the energy metabolic states of hepatic immune cells (16). Within the setting of sustained metabolic stress, the ability of immune cells to preserve coordination between their metabolic programs and functional states, and to readjust this relationship when necessary, represents the central basis of the immunometabolic adaptability discussed in this review.

In a healthy state, this adaptability is reflected in the capacity of immune cells to dynamically transition between functional states in response to changes in the tissue environment and external stimuli, while maintaining broad coordination among metabolic demands, functional output, and inflammatory regulation (17). This process involves more than a simple shift in metabolic mode or a reversible change in functional phenotype; rather, it also depends on whether metabolic support, functional transition, and response thresholds can remain relatively well coordinated under conditions of persistent stress. From a research perspective, changes in this coordinated state may be assessed by integrating the ability of immune cells to transition between functional states, glycolytic and oxidative metabolic phenotypes, and metabolic and immune indicators associated with the persistence or resolution of inflammation (18). On this basis, this review further discusses how impairment of this coordinating capacity may allow inflammatory responses to persist and contribute to tissue remodeling (19). By integrating current experimental findings and clinical evidence, this review seeks to establish a conceptual framework centered on immunometabolic imbalance, with the aim of providing insight into the limited translational efficacy of current MASH therapies and a theoretical basis for interventions targeting the gut–liver axis (20).

## 2 Gut microbial dysbiosis initiates hepatic immunometabolic priming

### 2.1 Functional metabolic shifts reshape a pro-inflammatory gut environment

Traditional microbiome studies have largely characterized alterations in gut microbial communities at the taxonomic level, such as fluctuations in the Firmicutes/Bacteroidetes ratio (21). However, in the pathological context of MASH, emerging evidence suggests that functional metabolic outputs generated by the microbiota may participate more directly in host pathological processes than taxonomic composition alone (7, 22). When the gut microbial ecosystem shifts from homeostasis to dysbiosis, its defining feature is not merely a simple change in the abundance of individual taxa, but rather a systemic alteration of the luminal metabolic landscape with pro-inflammatory characteristics (23).

Across multiple studies in patients with MASH and in relevant animal models, reduced abundance of butyrate-producing bacteria, such as *Faecalibacterium prausnitzii*, frequently coincides with decreased levels of short-chain fatty acids (SCFAs) (24). As an essential energy substrate for intestinal epithelial cells, reduced butyrate availability may alter epithelial energy metabolism and subsequently disrupt the synthesis and stability of tight junction proteins (25). This alteration rarely occurs in isolation; rather, it commonly coexists with the accumulation of inflammatory mediators, changes in bile acid composition, and diet-induced epithelial metabolic stress, collectively contributing to impaired intestinal barrier function (26).

Concurrently, an increased relative abundance of opportunistic pathogens, such as members of the Enterobacteriaceae family, is frequently accompanied by elevated endotoxin burden and accumulation of metabolic intermediates, including ethanolamine (27). These metabolites function not only as intermediates of microbial metabolism but may also further compromise intestinal barrier defenses by disrupting mucin layer integrity and activating Toll-like receptor–associated signaling pathways in epithelial cells (25). Collectively, these alterations increase the likelihood that pathogen-associated molecular patterns traverse the epithelial barrier and enter the portal circulation, thereby subjecting the liver to chronic exposure to low-grade yet persistent pro-inflammatory stimuli (7). Accordingly, MASH-associated alterations in the gut microbiota are more appropriately interpreted as a process of functional microenvironmental remodeling driven by changes in metabolic output, characterized by a shift from a state predominantly supporting nutritional metabolism toward one favoring pro-inflammatory signaling inputs, rather than as a mere disruption of microbial community structure (28).

### 2.2 Loss of protective microbial signals weakens hepatic receptor pathways

Under physiological conditions, gut-derived metabolites contribute to the maintenance of immune tolerance and metabolic homeostasis in the liver by activating specific nuclear or membrane receptors (29, 30). During the initiation and progression of MASH, reductions in metabolite availability, alterations in receptor expression profiles, or impairments in signal transduction efficiency may collectively weaken these regulatory mechanisms, thereby diminishing the liver’s capacity to buffer pro-inflammatory stimuli (31).

Reduced levels of SCFAs are associated with multiple immunoregulatory disturbances (32). Under homeostatic conditions, butyrate sustains the acetylation status of promoter regions of immunoregulatory genes such as *Foxp3* by inhibiting histone deacetylase (HDAC) activity, thereby supporting regulatory T cell differentiation and phenotypic stability (33). As SCFAs levels decline, this epigenetic regulatory effect may be attenuated, rendering pro-inflammatory transcriptional programs more readily activated (34). Moreover, SCFAs deficiency may compromise AMPK signaling, indirectly suppress autophagic flux and promoting intracellular accumulation of lipids and damaged mitochondria, ultimately exacerbating metabolic stress (35).

Alterations in bile acid composition represent another critical contributing factor (36). Gut microbiota dysbiosis can impair the conversion of primary to secondary bile acids, thereby modifying ligand availability for receptors such as the FXR and TGR5 (37). Attenuation of FXR signaling may relieve transcriptional repression of lipogenic genes and weaken its regulatory role in inflammatory control, rendering Kupffer cells more responsive even to relatively low-level stimuli (38). Meanwhile, intrahepatic accumulation of hydrophobic bile acids may compromise mitochondrial membrane stability and promote reactive oxygen species production, thereby exacerbating intracellular oxidative stress (39).

In addition, dysregulation of tryptophan metabolism is considered to contribute to the development of gut–liver axis dysfunction (40). Reduced production of indole-derived metabolites can attenuate signaling through the AhR, which plays a critical role in sustaining interleukin-22 (IL-22) production by intraepithelial immune cells and in promoting epithelial repair (41). Diminished AhR activity may further compromise barrier restoration capacity, thereby coinciding with increased intestinal permeability and enhanced influx of inflammatory signals (42).

### 2.3 Accumulated pro-inflammatory metabolites sustain immune stress

Within the MASH-associated metabolic milieu, the biological roles of certain metabolites may shift from supporting homeostatic maintenance toward modulating immune regulation, thereby altering the functional orientation of immune cells (23). This shift does not imply that metabolites directly determine immune cell fate; rather, they are more likely to constrain metabolic flexibility required for transitions between distinct functional states by reshaping the relative balance among intracellular metabolic pathways (43).

Aberrant accumulation of succinate and branched-chain amino acids (BCAAs) has frequently been reported to associate with enhanced pro-inflammatory immune responses (44). Under specific conditions, elevated intracellular succinate levels in macrophages can inhibit prolyl hydroxylase activity, thereby stabilizing hypoxia-inducible factor-1α (HIF-1α) and inducing a metabolic state resembling pseudohypoxia (45). This state is typically accompanied by increased glycolytic flux and augmented production of pro-inflammatory cytokines. Most supporting evidence, however, derives from animal models and *in vitro* studies, and both the magnitude and biological relevance of these effects may vary across hepatic immune cell subsets. Consequently, their applicability to human pathology remains insufficiently defined (46).

Meanwhile, elevated circulating BCAA levels may act as a nutrient signal to activate mechanistic target of rapamycin complex 1 (mTORC1), thereby supporting immune responses under conditions of high metabolic demand (47). In T cells, mTORC1 activation is commonly associated with enhanced glycolysis and a bias toward T helper 17 (Th17) differentiation, a process that may contribute to the persistence of inflammatory responses in specific metabolic contexts (48). Nevertheless, increased BCAA levels may also reflect broader systemic metabolic dysregulation, and current evidence remains insufficient to clearly define their causal direction or context-dependent roles across different stages of disease (49).

**Figure 1:**
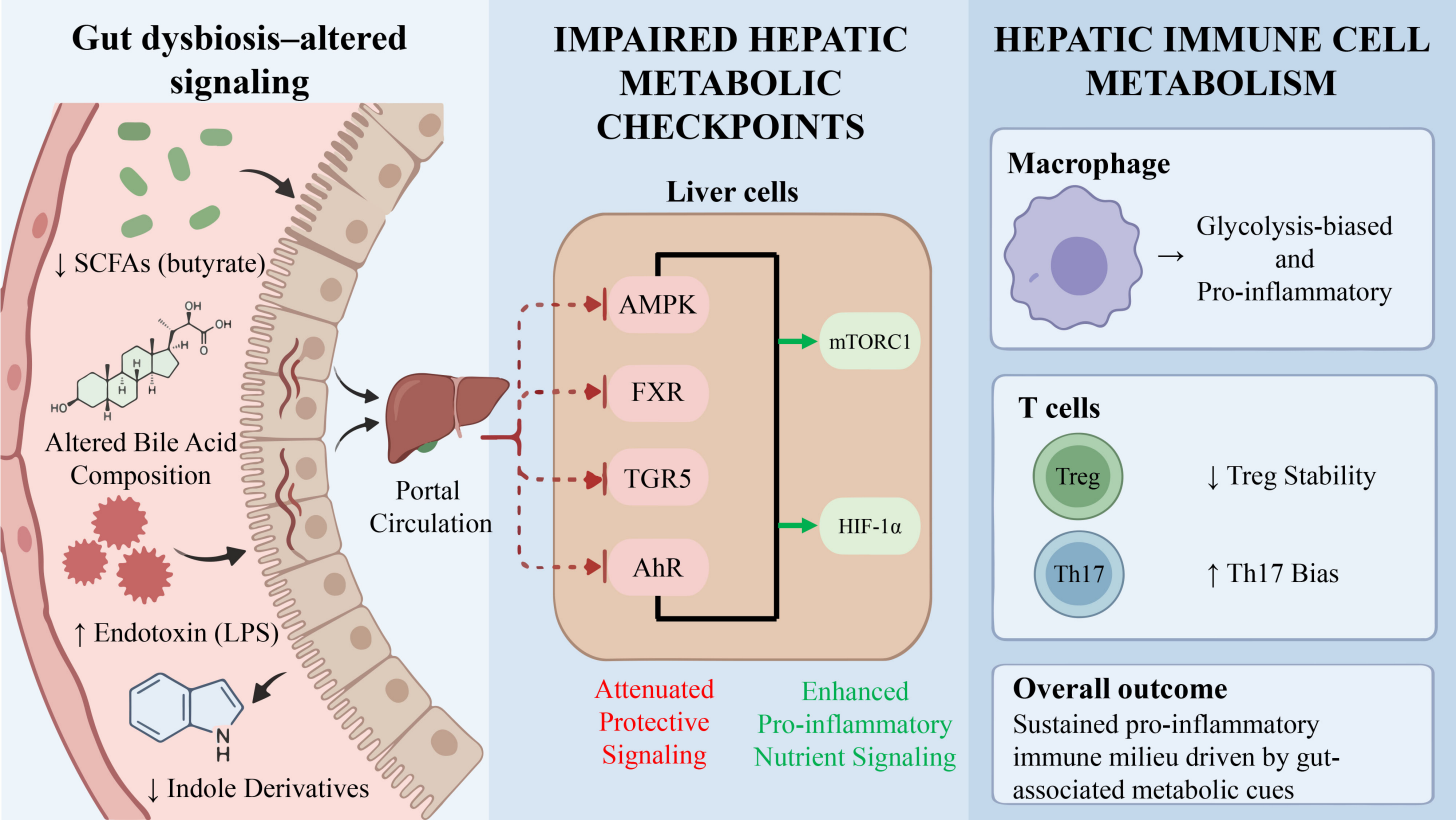
Gut-derived metabolic signals reprogram hepatic immune metabolism in MASH. Altered gut microbiota in MASH leads to reduced SCFAs, secondary bile acids, and tryptophan metabolites, and increased LPS, succinate, and BCAAs. These changes impair FXR, TGR5, AhR, and AMPK signaling in liver immune cells, promoting glycolysis-driven pro-inflammatory phenotypes in macrophages and Th17 expansion, while reducing Treg stability. The overall effect is loss of immunometabolic flexibility and enhanced hepatic inflammation.

Overall, coordinated alterations in multiple metabolic signals may collectively reprogram the metabolic state of hepatic immune cells, favoring the maintenance of pro-inflammatory metabolic programs while reducing sensitivity to inhibitory regulatory cues (14, 50). This metabolism-associated immunological shift provides a potential conceptual framework for understanding the persistence of inflammation in MASH and the limited efficacy of conventional anti-inflammatory interventions (51). A schematic overview of gut microbiota–derived metabolites in shaping hepatic metabolic checkpoints and immune tone is presented in **Figure 1**, and the corresponding metabolic signals, sensing pathways, and potential immunometabolic effects are summarized in **Table 1**.

**Table 1:** Gut-derived metabolic signals and their immunometabolic reprogramming effects in MASH.

Gut-derived metabolic signals	Major trend	Key sensing pathways/receptors	Primary cellular targets	Immunometabolic mechanisms
SCFAs (particularly butyrate)	↓	HDAC, AMPK	Reduced Treg stability; decreased autophagic flux; increased susceptibility to pro-inflammatory transcription	Attenuated HDAC inhibition reduces *Foxp3* acetylation, impairing Treg stability; reduced AMPK activity suppresses autophagy and exacerbates metabolic stress
Secondary bile acids	↓	FXR, TGR5	Weakened anti-inflammatory transcriptional regulation; reduced inhibition of inflammasome activity	Attenuation of anti-inflammatory signaling and loss of cAMP-dependent inhibition of NLRP3 inflammasome assembly
Succinate	↑	PHD–HIF-1α	Stabilization of HIF-1α; enhanced glycolysis; increased IL-1β expression	Inhibition of PHD stabilizes HIF-1α, induces pseudohypoxia, and promotes glycolysis-driven IL-1β production
BCAAs	↑	mTORC1	Support of high-metabolic-demand immune responses; promotion of Th17 differentiation	Activation of the mTORC1–HIF-1α axis supports anabolic metabolism and promotes Th17 differentiation and pro-inflammatory polarization
LPS	↑	TLR4–NF-κB	Inflammasome priming; enhanced macrophage responsiveness	Provides inflammasome priming signals and upregulates pro-inflammatory cytokine and chemokine expression
Indole-derived metabolites	↓	AhR	Impaired IL-22–mediated barrier repair and immune regulation	Reduced AhR signaling leads to decreased IL-22 production, compromising intestinal barrier repair and local immune tolerance

SCFAs, short-chain fatty acids; HSCs, hepatic stellate cells; BCAAs, branched-chain amino acids; PHD, prolyl hydroxylase.

## 3 Hepatic immunometabolic reprogramming amplifies inflammation

### 3.1 Nutrient-sensing checkpoint dysregulation drives Th17/Treg axis shift

**Figure 2:**
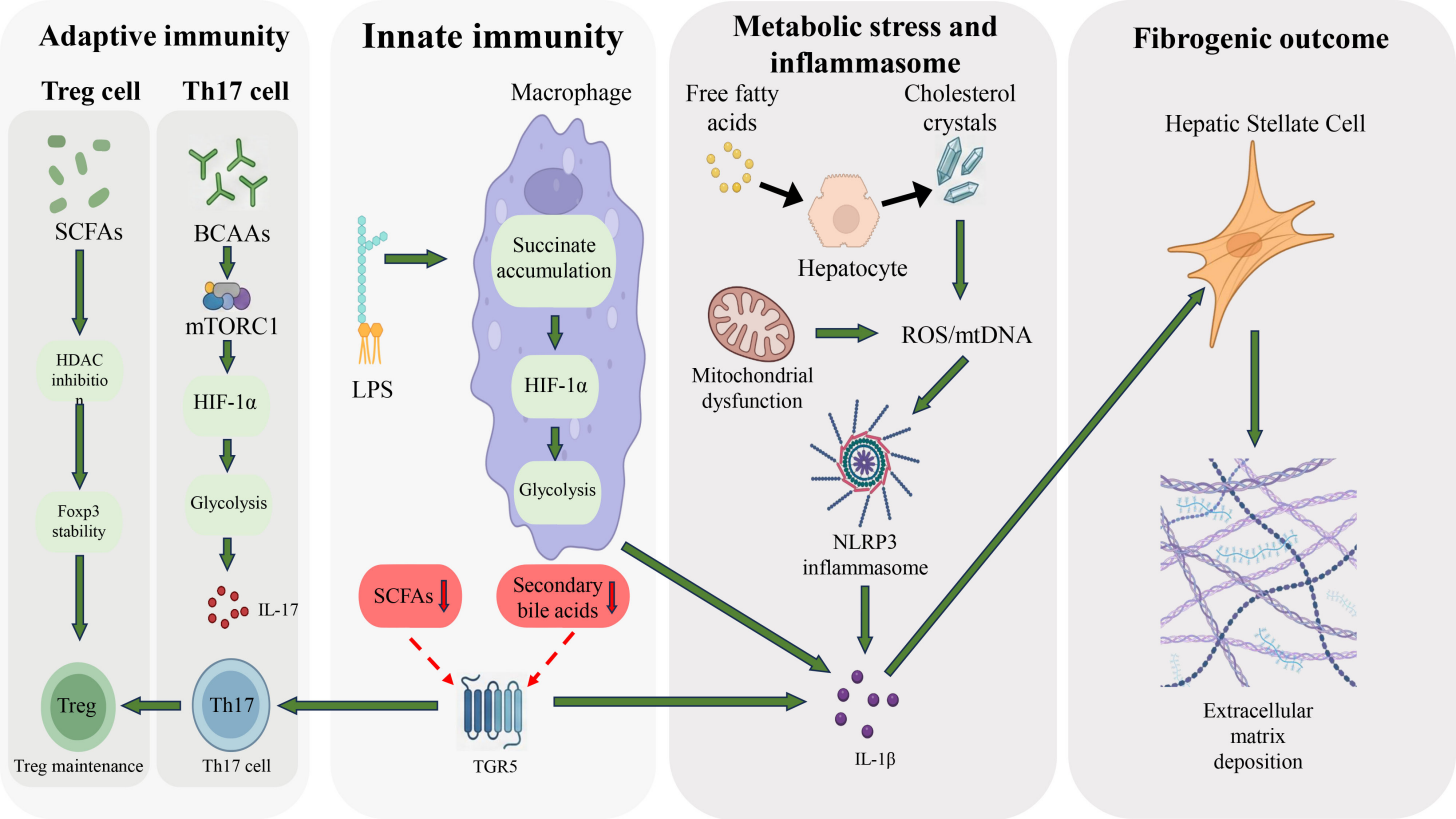
Mechanistic framework linking gut–liver axis dysfunction to NLRP3 inflammasome activation and fibrosis. Gut barrier injury and microbial translocation provide priming signals (LPS–TLR4), while metabolic stress (ROS, mtDNA, cholesterol) triggers NLRP3 activation in macrophages. Resulting IL-1β and IL-18 promote pyroptosis and hepatic stellate cell activation, leading to fibrosis. A feed-forward loop forms as IL-1β further damages the gut barrier, sustaining inflammation.

Disruption of hepatic immune homeostasis is commonly regarded as a critical inflection point in the progression of MASH from simple steatosis to inflammatory and fibrotic stages (52). Accumulating evidence suggests that imbalance in immunometabolic homeostasis constitutes a key transitional node in this process (**Figure 2**). Notably, shifts in the balance among helper T cell subsets—particularly changes in the relative proportions of Th17 cells and regulatory T cells (Treg)—are frequently observed as a representative phenotypic feature of alterations in the hepatic immune microenvironment (53). Under homeostatic conditions, Treg cells maintain immune tolerance through Foxp3-driven transcriptional programs and the secretion of anti-inflammatory mediators such as interleukin-10 (IL-10), whereas Th17 cells primarily participate in protective inflammatory responses (54). The metabolic dysregulation and chronic inflammatory milieu associated with MASH may, through the convergence of multiple factors, perturb this balance and facilitate the persistence of pro-inflammatory responses (55).

T-cell functional states are closely linked to their intracellular energy metabolic programs, and this regulatory layer may be regarded as an important metabolic checkpoint (56). Phenotypic stability of Treg cells typically depends on fatty acid oxidation (FAO) and AMPK-related signaling (57). As discussed above, in the setting of MASH-associated gut microbiota dysbiosis, reduced SCFAs availability may weaken HDAC/Foxp3-related epigenetic support and AMPK-associated metabolic maintenance, thereby compromising Treg phenotypic stability (58). In the T-cell context, the more important consequence of this change is the weakening of the buffering capacity of Treg cells against a persistent inflammatory milieu, thereby making the Th17/Treg balance more prone to shift toward a pro-inflammatory state (59).

Overall, alterations in the Th17/Treg ratio are more likely to reflect constraining effects of the metabolic milieu on T cell functional states rather than direct determination of cell fate by individual metabolic signals (60). Under specific conditions, IL-17–mediated amplification of inflammatory responses can promote neutrophil recruitment and influence activation of hepatic stellate cells through modulation of the local paracrine environment, thereby contributing to fibrosis-associated processes (61). However, the magnitude and persistence of these effects remain subject to coordinated regulation by multiple local and systemic factors (62).

### 3.2 Macrophage metabolic reprogramming favors pro-inflammatory bias

As a central component of the hepatic innate immune network, macrophages—including resident Kupffer cells and monocyte-derived infiltrating macrophages—play a pivotal regulatory role in the initiation and maintenance of inflammation in MASH (63). Although hepatocytes and liver sinusoidal endothelial cells are also exposed to substantial metabolic stress during disease progression, macrophages occupy a strategic intersection between microbial signal sensing, metabolic information integration, and amplification of inflammatory signaling (64). Consequently, they exert a prominent integrative influence on the establishment and persistence of immunometabolic imbalance. Moving beyond the classical M1/M2 polarization paradigm, current research reveals that hepatic macrophages span a continuous spectrum of marked functional heterogeneity. Their state transitions are thus better conceptualized as being driven by coupled metabolic programs and transcriptional regulation (65).

In the macrophage context, the sustained input of the gut-derived or systemic pro-inflammatory metabolic signals described above is more likely to promote the maintenance of a pro-inflammatory metabolic bias (66). Metabolic abnormalities represented by succinate accumulation generally indicate a greater tendency toward glycolysis-dominant programs and are commonly accompanied by increased expression of pro-inflammatory mediators such as IL-1β and TNF-α (67). While this state favors the persistence of inflammatory responses, it may also limit the transition of macrophages toward reparative functional states (68).

By contrast, macrophage functions associated with anti-inflammatory activity and tissue repair generally require more stable support from oxidative metabolism (69). In the setting of MASH, attenuation of the protective gut-derived metabolic signals described above may reduce the efficiency of activating anti-inflammatory transcriptional programs, thereby weakening repair-associated functional output (70). Importantly, this functional bias does not imply that macrophages are irreversibly fixed in a given state, but more likely reflects a reduction in the metabolic adaptive capacity required for transitions between distinct functional programs (71).

In addition, persistent metabolic stimulation may induce relatively durable metabolic and epigenetic remodeling in subsets of macrophages, leading to heightened inflammatory responsiveness upon subsequent challenges (72). Emerging evidence suggests that epigenetic mechanisms, including histone modifications, may contribute to this process (73). However, the extent, duration, and reversibility of such phenomena in human MASH pathology remain insufficiently defined, and further investigation is required to substantiate their relevance (74).

### 3.3 Metabolic stress lowers the threshold for inflammasome activation

The inflammatory phenotype of MASH is not driven by a single immune stimulus, but rather arises from reciprocal amplification between metabolic stress and immune activation signals (75). Prolonged exposure of hepatocytes to excess free fatty acids and cholesterol can induce lipotoxic responses, characterized by mitochondrial dysfunction, increased production of reactive oxygen species (ROS), and sustained accumulation of intracellular stress signals (76). Mitochondrial DNA (mtDNA) and oxidized lipids released from damaged mitochondria can act as damage-associated molecular patterns (DAMPs), which are sensed by neighboring immune cells and thereby potentiate local inflammatory responses (77).

Against this backdrop of metabolic stress, the NLRP3 inflammasome, as a molecular platform linking metabolic perturbations to inflammatory responses, has been shown to exhibit enhanced activity in multiple experimental models (78). Cholesterol crystals, ROS, and signals associated with mitochondrial damage can all promote assembly of the NLRP3 complex and induce the maturation and release of IL-1β and interleukin-18 (IL-18) (79). At the same time, attenuation of the inhibitory metabolic restraints that normally support homeostasis may also render the inflammasome more prone to remain in a heightened reactive state under conditions of persistent metabolic stress (80).

Persistent sterile inflammatory signals not only act on immune cells but also influence the functional state of hepatic stellate cells through multiple pathways (81). Reactive oxygen species, mediators released from apoptotic or pyroptotic hepatocytes, and Th17–associated cytokines, such as interleukin-17 (IL-17), collectively contribute to the establishment of a pro-fibrotic microenvironment and are linked to extracellular matrix deposition (82). It should be noted, however, that this process of inflammatory amplification is unlikely to be confined to inflammasome signaling alone (83). In the setting of concomitant mitochondrial injury and metabolic stress, DAMPs, including mtDNA, may be sensed by neighboring immune cells, while gut-derived LPS-TLR4 signaling may provide sustained priming cues for hepatic innate immune cells (84). At the same time, direct inflammatory responses in other hepatic cell types may also contribute to shaping the local inflammatory milieu (85). Accordingly, inflammation in MASH may be more appropriately understood as the result of coordinated interactions among metabolic stress, mitochondrial damage, and multiple immune sensing pathways, with the NLRP3 inflammasome serving more plausibly as a central integrative and amplifying platform rather than the sole pathway (86).

### 3.4 Genetic risk variants exacerbate immunometabolic maladaptation

Beyond exogenous metabolic burden and inflammatory signals, host genetic background plays an equally important role in shaping hepatic sensitivity to metabolic stress (87). Genome-wide association studies have identified multiple genetic variants associated with an increased risk of MASH progression, among which patatin-like phospholipase domain–containing protein 3 (PNPLA3) I148M and transmembrane 6 superfamily member 2 (TM6SF2) E167K are the most representative (88). These variants commonly disrupt lipid droplet remodeling and very-low-density lipoprotein secretion in hepatocytes (89), thereby establishing a hepatic metabolic context that is more permissive to lipid accumulation (90). Within an immunometabolic framework, the significance of these genetic variants lies less in directly determining inflammatory outcomes than in shaping the baseline hepatic response to sustained metabolic stress, thereby influencing the ability of immune cells to maintain coordination between functional states and metabolic programs.

Within this framework of genetic susceptibility, metabolic and inflammatory signals along the gut–liver axis may be further amplified (91). For example, the PNPLA3 I148M variant impairs lipid droplet hydrolytic capacity, rendering hepatocytes more prone to lipid retention and, under inflammatory conditions, to heightened oxidative and endoplasmic reticulum stress responses (92). Beyond intracellular lipotoxicity, the aberrant metabolic state associated with lipid retention may also affect hepatocyte–immune cell crosstalk (93). Persistent lipid accumulation may increase the release of stress-associated lipid signals and may also alter cellular lipid composition, thereby reshaping the metabolically relevant cues present in the local microenvironment (94). Through these paracrine effects, Kupffer cells may become more sensitive to low-grade danger cues and metabolic stimuli, rendering them more prone to inflammatory activation even at relatively low levels of PAMPs or metabolic stress signals (95). Thus, PNPLA3-associated lipid retention may contribute not only to hepatocellular lipotoxic stress, but also to amplified innate immune activation by altering hepatocyte–Kupffer cell signaling coupling (96). By contrast, the TM6SF2 E167K variant appears to act primarily by impairing very-low-density lipoprotein secretion, thereby aggravating intracellular lipid retention in hepatocytes and increasing intrinsic metabolic stress (97). In this setting, its effect may not necessarily be reflected as sustained activation of a specific inflammatory pathway, but is more likely to increase hepatic sensitivity to gut-derived metabolic and inflammatory signals, thereby creating a permissive background for the establishment of Kupffer cell priming, the maintenance of a pro-inflammatory metabolic bias, and a greater propensity for inflammatory amplification (98).

Accordingly, within the framework discussed here, the development and progression of MASH may be more appropriately understood as the result of the combined effects of genetic susceptibility and gut–liver axis–related metabolic stress (99). Genetic variants may not directly determine whether inflammatory responses occur, but they can increase hepatocellular lipotoxic burden, enhance hepatic susceptibility to gut-derived metabolic and inflammatory signals, and create a permissive background in which hepatic innate immune cells are more likely to enter a primed or pro-inflammatory state. In doing so, they may reduce the capacity of immune cells to maintain, or re-establish, coordination between functional states and metabolic programs under sustained metabolic stress (88). In this context, genetic risk is better understood as an important background factor that shapes the baseline of immunometabolic adaptability, rather than as an independent module detached from the broader immunometabolic narrative.

## 4 NLRP3 inflammasome mediates immune-metabolic–fibrotic transition

### 4.1 Two-signal activation of NLRP3 by gut-derived and metabolic stressors

The NLRP3 inflammasome serves as one of the key molecular platforms within the innate immune system that links metabolic stress to inflammatory responses, and has been shown in multiple models of metabolic inflammation to participate in the amplification and maintenance of inflammatory signaling (100). During the progression of MASH, both experimental studies and clinical observations indicate that increased NLRP3 inflammasome activity correlates with inflammatory severity and disease progression (101). However, its role is more likely to reflect amplification and persistence of pre-existing inflammatory responses rather than acting as a single, deterministic pathogenic driver (102).

Activation of the NLRP3 inflammasome follows a tiered regulatory scheme, requiring both a transcriptional priming phase and subsequent stimuli that surpass the activation threshold (103). During the priming stage, impairment of intestinal barrier function can increase the portal influx of LPS into the liver (104). Engagement of TLR4 on Kupffer cells by LPS activates the nuclear factor-κB (NF-κB) signaling pathway, leading to upregulation of NLRP3 and the precursor forms of IL-1β and IL-18 (105). This process places cells in a sensitized, primed state rather than directly triggering inflammasome assembly (106).

In the subsequent activation phase, intracellular metabolic and structural stress signals play a more decisive role (107). Excess saturated fatty acids, accumulation of cholesterol crystals, or mitochondrial dysfunction are often accompanied by reduced lysosomal membrane stability and increased generation of ROS (108). Oxidized mtDNA and ROS released from damaged mitochondria can, under specific conditions, act as intracellular danger signals that promote assembly of the NLRP3 complex and induce caspase-1 activation (109). Activated caspase-1 mediates maturation and secretion of IL-1β and IL-18, while simultaneously cleaving gasdermin D (GSDMD) to form membrane pores, thereby inducing pyroptosis-like cell death (110). DAMPs, such as ATP and high-mobility group box 1 (HMGB1), released during pyroptosis can further potentiate local inflammatory signaling and sustain the amplification of inflammation (111).

It should be emphasized that, although the molecular events described above are supported by relatively consistent evidence across multiple experimental models, their quantitative contributions may vary substantially across different stages of MASH and among distinct patient populations (112, 113). An overview of the associated molecular events and regulatory nodes is summarized in **Table 2**.

**Table 2:** Metabolic stress–driven mechanisms of NLRP3 inflammasome activation in MASH.

Stage	Key stimuli	Molecular events	Immunological consequences
Priming	LPS, TNF-α	NF-κB activation mediated by TLR4/TNFR, leading to increased transcription of NLRP3, pro-IL-1β, and pro-IL-18	Upregulation of inflammasome components and establishment of a highly responsive, primed immune state
Activation	ROS, cholesterol crystals, saturated fatty acids, mtDNA	Lysosomal damage and mitochondrial dysfunction; assembly of the NLRP3 complex and activation of caspase-1	Conversion of metabolic and structural stress signals into inflammatory responses
Execution	Activated caspase-1	Cleavage of GSDMD and formation of plasma membrane pores	Maturation and release of IL-1β and IL-18; induction of pyroptosis and amplification of inflammatory signaling
Dysregulation	Secondary bile acid deficiency	Attenuation of the TGR5–cAMP axis, resulting in loss of inhibitory control over NLRP3 ubiquitination and assembly	Sustained inflammasome overactivation, promoting chronic inflammation and fibrotic progression

LPS, lipopolysaccharide; ROS, reactive oxygen species; mtDNA, mitochondrial DNA; GSDMD, gasdermin D.

### 4.2 Inflammasome-driven cytokines promote HSC activation and fibrogenesis

Hepatic fibrosis represents a central pathological feature in the progression of MASH, with hepatic stellate cells (HSCs) serving as the principal effector cell type (114). Under conditions of persistent inflammation and metabolic stress, HSCs undergo a gradual transition from a quiescent state characterized by vitamin A–rich lipid droplets to a myofibroblast-like phenotype with enhanced proliferative, contractile, and extracellular matrix–secreting capacities (14, 115). This phenotypic conversion is not driven by a single signaling pathway, but rather arises from the coordinated actions of multiple immune mediators, metabolic alterations, and intracellular transcriptional regulatory programs (16, 116).

Paracrine signals derived from activated Kupffer cells and other immune populations play a critical regulatory role in HSC activation. For example, IL-1β promotes HSC proliferation and upregulates expression of tissue inhibitor of metalloproteinases 1 (TIMP1), thereby influencing the regulation of extracellular matrix turnover (117, 118). Transforming growth factor-β (TGF-β), in turn, induces transcription of α-smooth muscle actin (α-SMA) and type I collagen through the Smad2/3 signaling axis, driving sustained deposition of fibrotic matrix (119). Concurrently, HSCs undergo pronounced metabolic adaptations during functional state transitions to accommodate increased energetic and biosynthetic demands (120).

During the transition from quiescence to activation, HSCs typically exhibit a metabolic shift characterized by enhanced glycolysis, accompanied by upregulation of hexokinase 2 (HK2) and pyruvate kinase M2 (PKM2) (121). This metabolic program supports the energetic and carbon source requirements of cell proliferation, cytoskeletal remodeling, and extracellular matrix synthesis (122). In addition, activation of glutamine metabolism can replenish tricarboxylic acid cycle intermediates, providing metabolic support for collagen biosynthesis and associated epigenetic regulatory processes (123). Vitamin A–containing lipid droplets stored in quiescent HSCs are progressively depleted during activation, a process linked to enhanced lipophagy, with released fatty acids supplying initial energy through β-oxidation to facilitate phenotypic conversion (122).

Beyond paracrine stimulation, HSCs can also directly sense gut-associated metabolic and inflammatory cues. Expression of TLR4 and the FXR enables HSCs to respond to LPS and alterations in bile acid composition (124). LPS signaling through the TLR4–myeloid differentiation primary response protein 88 (MyD88)–NF-κB axis induces chemokine expression, such as CCL2, thereby contributing to inflammatory cell recruitment (125). In parallel, reductions in secondary bile acids resulting from gut microbiota dysbiosis may attenuate FXR-mediated anti-fibrotic transcriptional regulation, diminishing endogenous restraint on HSC activation (126). Collectively, these changes accompany sinusoidal endothelial capillarization and hepatic architectural remodeling (116).

### 4.3 Feedback loop between inflammasome and gut barrier aggravates MASH

During the progression of MASH, impairment of intestinal barrier function, inflammasome activation, and fibrotic responses are more likely to form mutually reinforcing amplification loops rather than linear, unidirectional causal relationships (127). Increased intestinal permeability permits sustained translocation of microbial-associated molecules, such as LPS, into the portal circulation, thereby enhancing NLRP3 inflammasome–related priming signals in the liver (128). Concurrently, inflammasome-mediated release of IL-1β has been suggested in some studies to further compromise intestinal barrier integrity by modulating the expression of epithelial tight junction proteins, establishing a positive feedback loop that perpetuates pro-inflammatory signaling (129).

Within the hepatic microenvironment, activated hepatic stellate cells secrete chemokines, such as CCL2, promoting recruitment of circulating monocytes to the liver and their differentiation into inflammation-associated macrophages (130). Under persistent metabolic stress and microbial stimulation, these cells may undergo renewed activation of inflammasome-related pathways, thereby reinforcing the reciprocal interplay between inflammatory responses and fibrogenesis (131). As disease progression continues, impairment of bile secretion and increasing disturbances in intestinal bile acid composition further weaken protective signaling mediated by the FXR and TGR5, diminishing the host’s metabolic capacity to restrain these pathological processes (38, 132).

Overall, fibrotic progression in MASH is not driven by a single signaling pathway in isolation, but more likely emerges from dynamic interactions among gut–liver axis–associated metabolic dysregulation, amplification of inflammasome signaling, and imbalance in tissue repair processes (127). This integrative perspective helps explain why interventions solely targeting inflammatory pathways often fail to reverse established fibrosis (133), and further suggests that strategies aimed at improving intestinal barrier integrity and restoring metabolic homeostasis may serve as complementary approaches to limit disease progression (128).

## 5 Therapeutic insights: targeting the gut–liver immunometabolic axis

### 5.1 Functional microbiota modulation to restore barrier and metabolite output

With the deepening understanding of immunometabolic mechanisms underlying MASH, gut microbiota–based intervention strategies have gradually shifted from early nonspecific supplementation toward precision modulation aimed at functional restoration (134). Increasing emphasis is being placed on improving intestinal barrier integrity and re-establishing the homeostatic output of key microbial metabolites (135). To this end, next-generation probiotics (NGPs) proposed in recent years have emerged as targeted candidates for microbiota interventions (136).

For example, *Akkermansia muciniphila* has been associated in certain animal models and early human studies with improvements in intestinal mucus layer architecture and phenotypes related to barrier integrity, thereby reducing the risk of translocation of microbial-associated molecules into the portal circulation (137). In parallel, intervention strategies targeting butyrate-producing bacteria aim to enhance local production of SCFAs, supporting the energetic requirements of intestinal epithelial cells and maintenance of barrier function, and potentially modulating epithelial gene expression through epigenetic mechanisms (138). It should be noted, however, that intervention outcomes vary substantially depending on strain composition, dosing regimens, and host baseline characteristics, and that long-term safety and reproducibility of these effects require validation in higher-quality population-based studies (139).

Fecal microbiota transplantation (FMT) offers the potential advantage of rapidly reconstituting a more diverse gut microbial ecosystem and, to some extent, restoring ecological production of key metabolites such as secondary bile acids, thereby influencing the basal activity of receptor signaling pathways including the FXR and TGR5 (140). Nevertheless, FMT faces practical challenges related to donor–recipient matching, infection risk management, long-term engraftment stability, and regulatory standardization (141). Against this backdrop, defined and more controllable synthetic consortia, as well as microbiota modulation strategies focused on metabolite restoration, may hold greater promise for future clinical translation (142).

Given that the efficacy of microbiota interventions is highly susceptible to factors such as dietary patterns, concomitant medications (particularly antibiotics and glucose-lowering agents), geographic variation, and individual baseline microbiota composition, future studies should systematically account for these confounders (143). Moreover, comprehensive evaluation of intervention outcomes should incorporate objective measures such as engraftment stability and metabolite output to more accurately assess therapeutic potential (144).

### 5.2 Receptor and metabolic pathway targeting for immune rebalancing

Given the close interplay between gut–liver axis–associated metabolic signaling and immune regulation, pharmacological interventions targeting key receptor pathways have emerged as an important avenue in MASH therapeutic development (145). Farnesoid X receptor (FXR) agonists, such as obeticholic acid, induce downstream transcriptional programs including small heterodimer partner (SHP), thereby modulating the expression of genes involved in lipid metabolism and inflammation (146). Some studies suggest that these agents may be associated with improvements in hepatic histological parameters to a certain extent; however, the magnitude of efficacy, the spectrum of suitable patient populations, and the profile of adverse effects require careful balancing through more comprehensive clinical evaluation (147).

Agonist-based strategies targeting TGR5 aim to exert inhibitory regulation over inflammasome-related pathways through signaling axes such as cyclic AMP–protein kinase A (cAMP–PKA) (148). However, clinical evidence supporting these approaches remains limited, and their potential tissue-specific effects as well as safety liabilities have yet to be fully elucidated (149). Consequently, the practical therapeutic value of TGR5-targeted strategies in MASH cannot yet be definitively determined (150).

Beyond receptor-mediated pathways, cellular energy-sensing nodes have also been proposed as potential intervention points to enhance immunometabolic regulation (151). Agonists of AMPK or inhibitors of mTORC1 may, at least mechanistically, reduce metabolic stress in immune cells by improving autophagic flux and promoting clearance of damaged mitochondria and excess lipids, thereby decreasing the likelihood of sustained pro-inflammatory activation (152). Importantly, immune cell functional states do not conform to a simple dichotomy between inflammatory and reparative programs; rather, these agents are more likely to act by modulating the dynamic balance among multiple functional states than by inducing linear state transitions (153).

In addition, intervention strategies targeting tryptophan metabolism and the AhR have been proposed to support epithelial repair and IL-22–associated immune functions. Nevertheless, current evidence remains insufficient to draw firm conclusions regarding their clinical efficacy, appropriate patient populations, or long-term safety in MASH, and further investigation is required to substantiate their therapeutic relevance (154).

### 5.3 Precision strategies guided by immunometabolic profiling

MASH exhibits pronounced heterogeneity with respect to disease stage, inflammatory severity, rate of fibrotic progression, and gut microbial background, a feature widely regarded as a major contributor to variability in clinical trial outcomes (23, 155). In clinical practice, such heterogeneity cannot be readily explained by the degree of steatosis or conventional metabolic indices alone (156). Emerging evidence suggests that the capacity of the hepatic immune system to adapt to metabolic stress may, at least in part, shape disease trajectories (157). Importantly, the concept of “immunometabolic adaptability” provides a framework for understanding why individuals with similar metabolic risk profiles may nonetheless experience divergent disease outcomes (158). This heterogeneity is not merely a reflection of differences in disease severity along a continuous spectrum, but may also indicate that the dominant immunometabolic drivers are not entirely the same across different stages of disease.

In some patients, impaired intestinal barrier integrity and sustained translocation of microbial-associated molecules may represent dominant features, placing hepatic immune cells in a chronic, low-grade primed state that lowers the threshold for inflammatory activation (159). By contrast, other patients may be primarily driven by endogenous lipotoxicity, mitochondrial dysfunction, and metabolic stress, with inflammatory responses more closely linked to the accumulation of sterile danger signals (160). Despite differences in upstream triggers, both trajectories may converge during disease progression on reduced metabolic flexibility of immune cells, rendering pro-inflammatory states difficult to resolve and facilitating progression toward inflammatory and fibrotic stages (161).

From a clinical assessment perspective, conventional biochemical markers and histological scoring systems primarily capture end-stage manifestations of hepatic injury and are poorly suited to reflect the dynamic metabolic states of immune cells (162). In contrast, metabolic signals related to gut microbial function and immunometabolic status may, at the research level, more sensitively indicate differences in disease-driving mechanisms (101). For instance, serum or fecal levels of short-chain fatty acids, bile acid composition, succinate abundance, or endotoxin-related markers can partially reflect variations in metabolic input along the gut–liver axis (163). Moreover, inflammasome-associated mediators, such as IL-1β and IL-18, may not only indicate inflammatory intensity but also signal insufficient resolution of inflammatory responses (164). It should be noted, however, that these markers are not yet suitable for routine clinical use and currently remain primarily research tools (101).

Building on this premise, the integration of multi-omics data to enable immunometabolic phenotyping has emerged as a research direction of considerable exploratory value (165). By combining metagenomic profiles reflecting microbial functional capacity, metabolomic fingerprints derived from serum or fecal samples, and immune phenotypic information, it may be possible to identify dominant pathological features across distinct patient subgroups (166). For example, in patients characterized primarily by intestinal barrier dysfunction and enhanced endotoxin-related signaling, interventions focused on barrier restoration and microbiota functional remodeling may be more appropriate (167). Conversely, in settings dominated by endogenous lipotoxicity, mitochondrial stress, and inflammasome pathway activation, pharmacological modulation targeting key metabolic and immune regulatory nodes such as the FXR, mTOR, or AMPK may better align with underlying pathophysiology (168).

Combination strategies guided by patient phenotypes also hold exploratory value at the research level (169). For instance, integrating approaches aimed at restoring barrier integrity with modulation of receptor pathways or metabolic nodes may simultaneously reduce upstream pro-inflammatory inputs and attenuate intrahepatic inflammatory amplification (103). Nevertheless, the rationale for such combination regimens depends on clearly defined mechanistic complementarity, appropriate selection of disease stage, and systematic safety evaluation (170). To facilitate comparison of therapeutic targets, mechanistic distinctions, and progress in clinical translation across different intervention strategies (171), the principal immunometabolic approaches targeting the gut–liver axis are summarized in **Table 3**.

**Table 3:** Immunometabolic intervention strategies targeting the gut–liver axis in MASH.

Intervention category	Primary targets/approaches	Immunometabolic regulatory effects	Evidence stage and limitations
Microbiota functional modulation	NGPs (e.g., *A. muciniphila*)	Improvement of mucus layer structure and tight junction integrity, reducing LPS translocation and indirectly attenuating hepatic immune priming	Animal models and early clinical studies; strong strain specificity and limited long-term engraftment
	Engineered butyrate-producing bacteria/synthetic consortia	Targeted enhancement of SCFAs production to support epithelial energy metabolism and reinforce immune tolerance–associated signaling	Preclinical stage; ecological safety, dose control, and regulatory feasibility remain unclear
Barrier- and metabolite-oriented approaches	FMT	Short-term restoration of microbial diversity and bile acid metabolic output, modulating basal FXR/TGR5 signaling	Limited clinical evidence; donor–recipient matching, infection risk, and long-term controllability remain constrained
Nuclear and membrane receptor targeting	FXR agonists	Regulation of bile acid and lipid metabolism and attenuation of inflammation-related transcriptional programs via SHP	Clinical trial stage; marked heterogeneity in efficacy and dose-related adverse effects
	TGR5 agonists	Activation of the cAMP–PKA axis and inhibitory modulation of inflammasome-associated signaling in Kupffer cells	Insufficient clinical evidence; tissue specificity and safety profiles require further evaluation
Metabolic checkpoint modulation	AMPK agonists/mTORC1 inhibitors	Improvement of autophagic flux and mitochondrial quality control, restoring immune cell metabolic flexibility and reducing pro-inflammatory bias	Strong mechanistic support; MASH specificity and long-term safety remain to be established
Immunoepithelial signaling support	AhR-oriented metabolic modulation	Promotion of IL-22–associated epithelial repair and indirect enhancement of barrier function and immune homeostasis	Largely mechanism-based inference; clinical applicability and therapeutic window remain uncertain
Precision stratified strategies	Multi-omics–guided combination therapies	Phenotype-matched modulation of dominant immunometabolic drivers (e.g., barrier dysfunction–dominant vs. metabolic stress–dominant)	Dependent on robust biomarkers; clinical trial design is complex

### 5.4 Translational challenges and unmet needs

Although multiple interventions targeting the gut–liver axis have demonstrated potential benefits in preclinical models, their translation into clinical application faces substantial challenges (172). From an immunometabolic perspective, the limited efficacy of single-target interventions may reflect their inability to restore the metabolic flexibility required for immune cells to transition between distinct functional states (173). Therapeutic strategies directed at specific metabolic or inflammatory pathways often attenuate signal intensity but fail to reprogram established pro-inflammatory metabolic programs (174).

For instance, agents targeting bile acid signaling or lipid metabolism may be associated with histological improvement in some patients, whereas others derive limited benefit or experience adverse effects (175). Such variability may be attributable to inter-individual differences in gut microbiota composition, the magnitude of metabolic signal input, and baseline immunometabolic states of immune cells (176). Similarly, heterogeneous responses to microbiota interventions suggest that altering microbial composition alone, without concomitant modulation of host immunometabolic status, may be insufficient to disrupt established positive feedback loops between inflammation and fibrosis (140, 177).

It should be further emphasized that current studies of the gut microbiota in patients with MASH remain markedly heterogeneous, and different cohorts do not show fully consistent patterns with respect to dominant microbial shifts, metabolomic features, or their associations with disease severity. Such variability may be related to differences in dietary patterns, medication exposure, geographic background, sample type, and analytical methodology, and it also underscores the need for caution when inferring unified mechanisms directly from cross-sectional associations (156). At the same time, early MASLD and MASH with established inflammation and fibrosis may not share the same dominant drivers; the former may be more strongly characterized by intestinal barrier dysfunction, metabolic priming, and lowered immune activation thresholds, whereas the latter may more prominently involve inflammatory amplification, tissue remodeling, and the maintenance of fibrosis (178). Likewise, the mixed results observed in interventions targeting the gut microbiota do not necessarily argue against the importance of mechanisms related to the gut microbiota, but may instead reflect multiple limitations, including differences in baseline microbial states, insufficient stability of microbial engraftment, mismatch between intervention and disease stage, and variation in endpoint selection (179).

In addition, interspecies differences impose practical limitations on mechanistic extrapolation. Rodents and humans differ substantially in bile acid pool composition, immune cell lineage distribution, and stability of gut microbial colonization, raising concerns that immunometabolic regulatory circuits observed in animal models may not be recapitulated in humans with comparable magnitude or configuration (180). At the same time, safety and tolerability represent critical constraints for systemic metabolic modulators. For example, potent FXR agonists have been associated with adverse events such as pruritus and alterations in lipid profiles, which may compromise long-term adherence and overall risk–benefit considerations (181).

Immunometabolic interventions are also likely to exhibit stage dependency. Available evidence suggests that their effects may vary across disease stages (98). In early disease, interventions aimed at improving intestinal barrier integrity and restoring protective metabolic outputs may help limit the establishment of immune priming states (26). By contrast, in established MASH and fibrosis, reducing metabolic burden alone may be insufficient to reverse disease progression; instead, combined modulation of inflammasome-related signaling and reprogramming of immune cell metabolic states may be required to counteract persistent inflammatory amplification (182). These hypotheses require systematic validation across distinct disease stages (183).

Accordingly, conceptualizing MASH as a disease of immunometabolic network dysregulation may help shift research paradigms from uniform intervention strategies toward stratified approaches based on immunometabolic features (184). Future clinical studies that incorporate parameters such as intestinal barrier status, metabolic signaling profiles, and degrees of immune activation at the design stage may enhance interpretability of efficacy outcomes and support the development of personalized interventions (185). At the same time, a balance must be maintained between suppression of inflammatory amplification and preservation of immune defense: while dampening inflammation can mitigate tissue injury, excessive immunosuppression may increase infection risk or impair essential tissue repair (115, 186). Consequently, future research should prioritize: (1) stage-appropriate endpoint selection, (2) patient stratification across multidimensional features spanning the microbiota, metabolism, and immunity, and (3) incorporation of companion biomarkers that are mechanistically relevant and quantifiable alongside systematic safety monitoring to narrow the translational gap between mechanistic evidence and durable clinical benefit.

## 6 Conclusion and perspective

MASH represents a critical intermediate stage in the progression of fatty liver disease toward cirrhosis and hepatocellular carcinoma (187). Its initiation and progression are not confined to the liver alone, but instead reflect a systemic immunometabolic imbalance involving regulation across multiple organs and biological levels (188). Accumulating evidence indicates that MASH does not arise from a linear sequence of discrete pathogenic events, but rather emerges from the interplay of multiple molecular and signaling pathways within a permissive metabolic context (46). In this regard, the concept of the gut–liver axis has substantially expanded our understanding of MASH pathogenesis, shifting the investigative focus from isolated hepatic injury toward an integrated network encompassing gut microbial ecology, inter-organ metabolic signaling, and immune regulation (189).

Current evidence suggests that alterations in the gut microbiota are not merely epiphenomena of MASH, but may influence the regulatory threshold of the hepatic immune microenvironment by reshaping the metabolite landscape (190). Importantly, this influence is more likely to manifest as modulation and amplification of inflammatory responses rather than acting as a single, deterministic causal driver (191). Within the integrative framework presented here, impaired intestinal barrier integrity and functional shifts in metabolic output may constitute early contextual features of gut–liver axis imbalance (163). Reductions in short-chain fatty acids, secondary bile acids, and indole-derived metabolites weaken inhibitory constraints on hepatic innate immunity, whereas elevations in succinate, branched-chain amino acids, and microbial-associated molecules can, under specific metabolic conditions, bias immune cells toward pro-inflammatory responsiveness (192).This immunometabolic shift accompanying changes in the metabolic milieu may constrain immune flexibility across functional states, thereby sustaining inflammasome activation and coinciding with fibrotic progression (193).

At the therapeutic level, interventions targeting the gut–liver axis offer important insights into both the understanding and management of MASH (194). Strategies ranging from restoration of intestinal barrier function and microbial homeostasis to modulation of immunometabolic states via nuclear receptors and energy-sensing pathways display potential mechanistic complementarity. Nonetheless, most approaches remain confined to preclinical studies or early-phase clinical trials, and their long-term efficacy and applicable patient populations have yet to be clearly defined (195). Interspecies differences, variability in microbial engraftment stability, and pronounced heterogeneity in metabolic and immune backgrounds among patients constitute substantial translational challenges, particularly limiting the reproducibility and predictability of microbiota interventions (196).

It should be emphasized that the reciprocal interactions between metabolic environments and immune functional states discussed here are not unique to MASH (197). Comparable immunometabolic reprogramming has been observed in other chronic metabolic inflammatory diseases (198). However, substantial differences in tissue microenvironments, cellular lineage composition, and metabolic constraints across diseases necessitate caution when extrapolating this framework (199). Viewing MASH as a representative model of systemic immunometabolic dysregulation may facilitate identification of shared regulatory principles from a comparative pathology perspective, but does not imply direct transferability of mechanisms to other disease contexts (200).

Looking forward, MASH research should move beyond predominantly correlative observations toward more targeted validation of key mechanistic pathways (201). Systematic integration of multi-omics approaches to delineate interactions among gut microbes, metabolic signals, and hepatic immune cells will help identify regulatory nodes that are truly operative within specific pathological contexts (202). Concurrently, patient stratification based on immunometabolic features holds substantial value for improving interpretability of clinical studies and understanding heterogeneity in therapeutic responses. Moreover, the development of *in vitro* models that more faithfully recapitulate the complex interactions of the human gut–liver axis may help overcome limitations inherent to animal models in immunometabolic regulation.

Overall, re-examining MASH from a systems immunometabolic perspective facilitates integration of disparate mechanistic evidence and deepens understanding of the intrinsic logic underlying persistent disease progression and limited therapeutic responsiveness. The integrative framework proposed here does not constitute a new pathogenic model, but rather serves as an analytical lens through which existing data and clinical heterogeneity can be interpreted. As insights into interactions between the gut microbiota and host immunometabolic regulation continue to advance, stratified investigations based on explicit mechanistic hypotheses centered on the gut–liver axis may provide more targeted avenues for improving long-term management of MASH.

## Glossary

AMPK: AMP-activated protein kinase
AhR: Aryl hydrocarbon receptor
BCAAs: Branched-chain amino acids
cAMP: Cyclic AMP
cAMP–PKA: Cyclic AMP–protein kinase A
DAMPs: Damage-associated molecular patterns
FAO: Fatty acid oxidation
FXR: Farnesoid X receptor
FMT: Fecal microbiota transplantation
GSDMD: Gasdermin D
HSCs: Hepatic stellate cells
HK2: Hexokinase 2
HMGB1: High-mobility group box 1
HDAC: Histone deacetylase
HIF-1α: Hypoxia-inducible factor-1α
IL-6: Interleukin-6
IL-10: Interleukin-10
IL-17: Interleukin-17
IL-18: Interleukin-18
IL-22: Interleukin-22
IL-1β: Interleukin-1β
LPS: Lipopolysaccharide
MASLD: Metabolic dysfunction–associated steatotic liver disease
MASH: Metabolic dysfunction–associated steatohepatitis
mTORC1: Mechanistic target of rapamycin complex 1
mtDNA: Mitochondrial DNA
MyD88: Myeloid differentiation primary response protein 88
NGPs: Next-generation probiotics
NLRP3: NLR family pyrin domain–containing 3
NF-κB: Nuclear factor-κB
PAMPs: Pathogen-associated molecular patterns
PNPLA3: Patatin-like phospholipase domain–containing protein 3
PPARγ: Peroxisome proliferator–activated receptor γ
PKM2: Pyruvate kinase M2
ROS: Reactive oxygen species
SCFAs: Short-chain fatty acids
SHP: Small heterodimer partner
TGR5: Takeda G protein–coupled receptor 5
Treg: Regulatory T cells
Th17: T helper 17
TIMP1: Tissue inhibitor of metalloproteinases 1
TLR4: Toll-like receptor 4
TGF-β: Transforming growth factor-β
TM6SF2: Transmembrane 6 superfamily member 2
TNF-α: Tumor necrosis factor-α
α-SMA: α-smooth muscle actin
